# A High-Throughput Assay for siRNA-Based Circadian Screens in Human U2OS Cells

**DOI:** 10.1371/journal.pone.0003457

**Published:** 2008-10-20

**Authors:** Christopher Vollmers, Satchidananda Panda, Luciano DiTacchio

**Affiliations:** 1 Regulatory Biology Laboratory, Salk Institute for Biological Studies, La Jolla, California, United States of America; 2 Heidelberg University Biochemistry Center (BZH), Heidelberg, Germany; Yale School of Medicine, United States of America

## Abstract

The advent of siRNA-based screens has revolutionized the efficiency by which functional components of biological processes are identified. A notable exception has been the field of mammalian circadian rhythms. Here, we outline a medium- to high-throughput siRNA-based approach that, in combination with real-time bioluminescence measurement of a circadian reporter gene, can be utilized to elucidate the effects of gene knockdown across several days in human cells.

## Introduction

Circadian rhythms are ∼24 h oscillations in behavior and physiology present in most organisms that evolved as an evolutionary response to the environmental changes brought upon by the day-light cycle [1], [2]. In mammals, the circadian system is composed of a collection of tissue-independent, peripheral oscillators and a central pacemaker present in the Suprahiasmatic Nuclei (SCN) of the hypothalamus. At the molecular level circadian rhythms originate on a genetic circuit formed by two interlocked transcription/translation feedback loops [3], [4]. In one of these loops, the bHLH-PAS transcription factors CLOCK and Bmal1 drive expression of the Period (*Per1* and *Per2*) and Cryptochrome (*Cry1* and *Cry2*) genes, whose protein products inhibit CLOCK/Bmal1 activity, thus producing ∼24 h rhythm in *Per* and *Cry* transcription. In the second one, CLOCK/Bmal1 drive transcription of *ROR* and *Rev-erb* class of nuclear hormone receptors, whose mutually-opposing action on the *Bmal1* promoter produces ∼24 h oscillations in transcription of the *Bmal1* gene. In all, the resulting oscillations in CLOCK/Bmal1 activity give rise to global circadian rhythmicity.

The circadian clock generates rhythmic transcription of up to 10% of the genome and modulates major functions of almost every organ [5], [6]. Indeed, circadian dysfunction, whether due to environmental or genetic factors, is associated with a plethora of ailments in both animal models and humans, including metabolism disorders, increased cancer incidence and abnormal aging [5], [7], [8], [9], [10], [11]. Furthermore, modern life circadian stressors, such as shift work and jetlag, are estimated to have an economic impact in the order of billions of dollars per year due to decrease productivity, injuries and absenteeism [12].

Because of the influence of the oscillator on key biological processes and its impact on human health and society, it has been the subject of intense research. A large part of this impetus has been directed towards understanding its molecular mode of action and, importantly, its molecular components [13], [14], [15], [16], [17]. However, as in other biological fields the discovery of new components based on classical genetic tools, like mutagenesis screens or candidate gene recombination-based disruption, is laborious and slow.

Within the past decade screening with focused and genome-scale RNAi libraries has revolutionized the efficiency and speed by which novel components in many biological processes are elucidated [18]. In contrast to other fields of biology, the use of large-scale siRNA screens to identify novel components of the circadian clock has not yet been achieved, primarily due to the difficulty in assay miniaturization with a compatible plate reader, lack of easily transfectable cell lines exhibiting robust circadian oscillations and technical challenges in maintaining constant temperature and cell culture conditions. We have overcome these challenges and developed a robust high throughput-compatible bioluminescence based assay that is amenable to siRNA based perturbation.

Rodent fibroblasts that are widely used for circadian cell-based assays [19], [20] typically exhibit very low transfection efficiencies. Therefore, we developed a human osteosarcoma U2OS cell reporter line (U2OS-B6) stably expressing a Bmal1 promoter-driven luciferase reporter construct (Bmal1::Luc) that exhibits robust circadian oscillations in bioluminescence. U2OS cells were selected because of their successful use in siRNA gene knockdown experiments in high-throughput assays using 384-well format [18], [21], [22]. These cells, when plated in 35 mm culture dishes and monitored with a 32-channel luminometer, exhibited robust bioluminescence rhythms that persisted for up to 7 days (data not shown). However, the relatively large culture dish size, longer integration time and limited number of channels make this format unsuitable for HTS based screens.

## Results

As a first step we set out to identify a temperature-controlled luminescence plate reader with kinetic measurements feature. We seeded 384-well plates with cells at varying concentrations between 2,500 and 40,000 cells per well in culture media (described below) containing 0.1 mM D-Luciferin and performed non-invasive kinetic bioluminescence measurement at 37°C. Of five different plate-readers tested, TECAN luminometers provided the best signal-to-noise ratio, temperature regulation and robustness in data acquisition. However, irrespective of luminometers, the adhesive optical tapes typically used to seal plates failed to maintain a proper seal after two days of data collection, introducing significant well-to-well variation in bioluminescence and/or cell survival. We found sealing the plates with custom-made glass coverslips (see Materials and Methods) and non-volatile “Dow Corning^R^ High Vacuum Grease” remarkably improved signal uniformity and cell survival. All cell density conditions exhibited almost equally robust oscillations (data not shown). Based on a review of the literature and on the recommendations of a number of manufacturers of siRNA transfection reagents, we chose to develop a protocol based on a density of 5,000 cells per well.

Next, we obtained a focused siRNA library comprising several known clock components. In this library, each gene is individually targeted by four different siRNAs. The logic of this approach is two-fold: First, it enables us to make a preliminary assessment of the significance of an effect based on whether it is observed with different siRNA oligonucleotides. Second, as it is impractical to validate the ability of each siRNA duplex to silence its target mRNA before carrying out the screen, targeting each gene four times enables us to prioritize hits for follow-up based on whether different siRNAs give rise to a specific phenotype.

**Figure 1:**
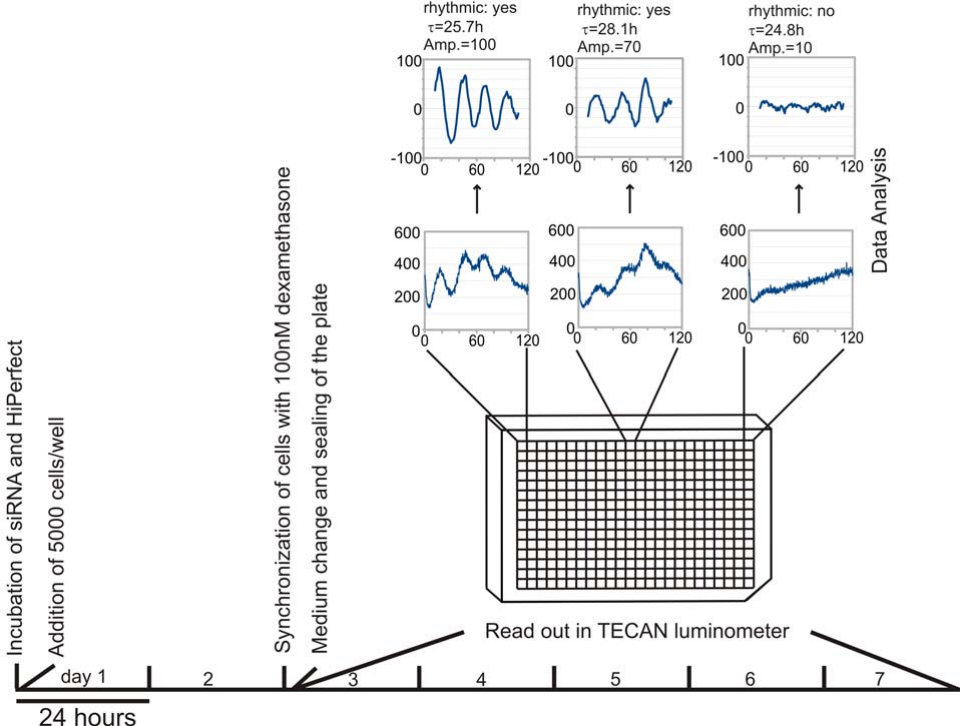
Schema of the kinetic 384-well siRNA assay timeline. Two days after transfection as described in the materials and methods, cells are shocked and subjected to continuous luminescence measurements for 5 days, following which the data is detrended and analyzed.

Each siRNA duplex was transfected in quadruplicate following a reverse-transfection protocol, as outlined in Figure 1. The data obtained for each well was detrended with a 24-h moving window average and smoothed using a 2-hour moving average. Period length and rhythmicity were determined by COSOPT analysis as previously described [23]. COSOPT evaluates rhythmicity through a statistical test, assigning a pMMC-beta value to each well. In this assay, robustly rhythmic wells had a value of 0, while arrhythmic ones were >0. The amplitude of each well's oscillations was defined as the difference between the 90th and 10th percentile.

After confirming the normality of the data by a Kolmogorov-Smirnov test, hits were defined based on three criteria: (a) Rhythmicity/arrhythmicity, (b) period length and (c) amplitude. The period length and amplitude of each quadruplicate set were tested for significant differences against 16 control wells (scrambled siRNA) with a two-tailed Student's t-test with α = 0.05. A positive hit was defined as a quadruplicate that is arrhythmic (>3 out of 4 wells show pMMC-beta value >0) or shows a significant difference (p<0.05) in amplitude or period length against the control.

Since statistical measures to evaluate a high throughput screen like the Z-factor [24] do not apply to our multidimensional readout, we defined the false positive rate (FPR). To achieve this, we utilized data from an entire plate transfected with scrambled siRNA to randomly assign 16 control wells and 92 quadruplicate “samples.” False positives were then defined as hits according to the criteria described above. Based on this, the average FPR of 25 independent randomizations was 10.13±2.4% (mean±sd), which is comparable to what other groups have reported [25], [26].

**Figure 2:**
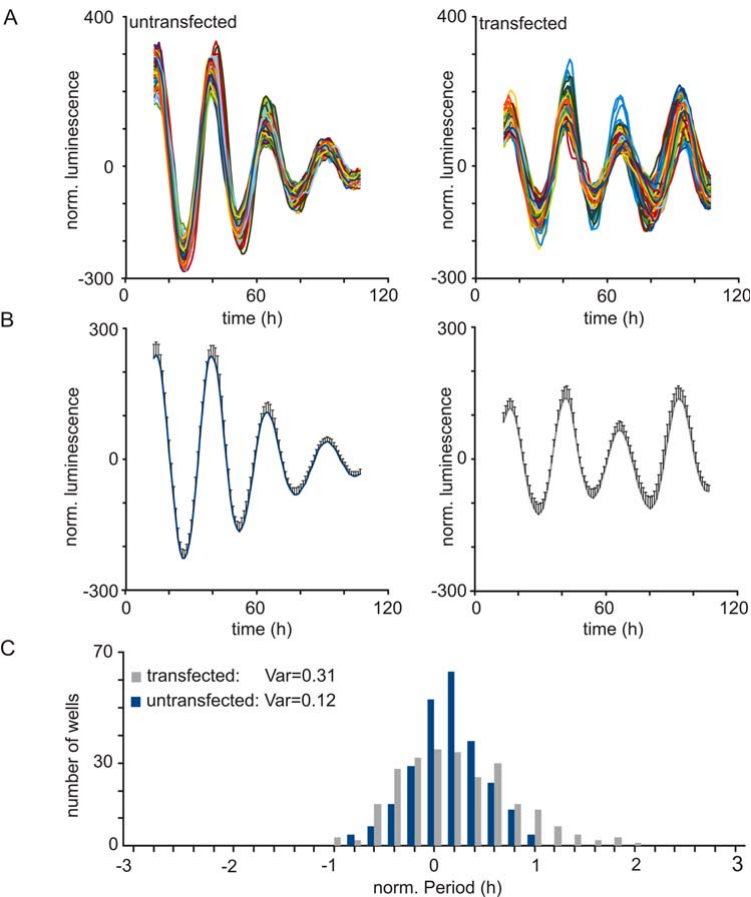
Effect of siRNA transfection on U2OS-B6 cells. (A) individual traces and (B) average (with s.d.) of detrended luminescence counts from wells with untransfected cells (left panel) or cells transfected with scrambled siRNA (right panel), n = 250 per condition. (C) Frequency distribution of the period length of both untransfected cells and cells transfected with scrambled siRNA shown in (A) and (B).

To assess the unspecific effects of siRNA transfection we compared the period length distribution between cells transfected with a scrambled siRNA and untransfected cells. Transfection with scrambled siRNA increased the variance of period length distribution (Figure 2C), but did not affect the robustness of single well oscillations (Figure 2A and B). Inter-plate variability in period length (25.82±0.97 h, n = 4) and amplitude (106±76, n = 4) was addressed by running scrambled siRNA transfected wells on every screening plate.

Next, the siRNAs for *Bmal1* and *Cry1* were tested for knockdown validation. These were chosen based on the clear phenotypes observed in immortalized cells derived from homozygote-null mutant mice [27], [28]. Specifically, *Bmal1* deficiency results in complete disruption of circadian oscillations, while *Cry1* disruption leads to a period shortening phenotype coupled to severe amplitude damping so that rhythmicity is lost in just over two days [27].

**Figure 3:**
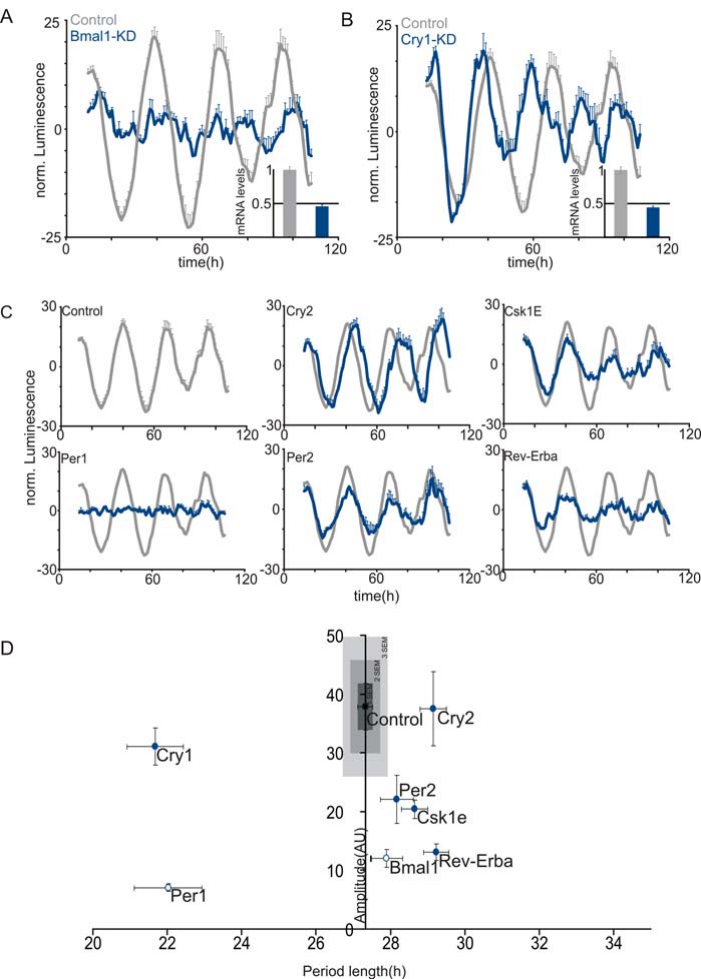
Assay validation. (A) Detrended luminescence traces of wells corresponding to either *Bmal1* (left, n = 4), *Cry1* (right, n = 4) or scrambled control siRNA (gray traces, both panels, n = 16). Relative mRNA (±s.d.) values were determined by Q-PCR (Inlets). (B) Detrended Luminescence traces of wells transfected with different siRNAs specific for the indicated circadian oscillator components (n = 4) or scrambled unspecific siRNA control wells (n = 16). (C) Amplitude and period length plots of the results obtained with siRNAs depicted in (a) and (b) (n = 4, s.e.m). Multiples of s.e.m. of the control siRNA (n = 16) are indicated in gray boxes. Arrhythmic wells are displayed as open circles.

Consistently, *Bmal1* knockdown led to circadian rhythm disruption and *Cry1* to a short-period, with dampened oscillations (Figure 3A and B). Q-PCR analysis of the siRNA-treated samples showed a decrease of ∼50% of the corresponding targeted transcripts levels compared to our non-specific controls 48 hours after transfection (Fig 3A and B, inlets). Finally, we also analyzed the effect of knocking down a small set of known oscillator components in this assay (Figure 3C and D). All the clock components tested were scored as hits according to these criteria. Importantly, circadian deficiencies observed in this 384 well based siRNA knockdown mimic results from siRNA based knockdown in 35 mm culture dishes, fibroblasts from the respective knockout mice, and behavioral rhythms in mice-carrying loss-of-function alleles of the respective genes [27], [29].

## Discussion

Until now the field of circadian biology has not fully benefited from the power of siRNA-based discovery of novel oscillator components. To address this, we have developed a method that is cost-effective, reliable and that will likely prove valuable to the study of the circadian oscillator for several reasons. First, this assay is medium- to high-throughput compatible, thus allowing for efficient and thorough screening for putative circadian clock genes. Second, the approach makes use of transfection of synthetic siRNA duplexes, circumventing the need for more complicated strategies, such those involving lentiviral or retroviral vectors. Third, this assay reveals clear circadian phenotypes caused by the knockdown of several clock components, some of which (*Bmal1*, *Cry2*, *Per1*) are similar to those observed in genetic-null murine models. However, not all the phenotypes we observed are identical to those of the corresponding knockout mouse cells. For example, in this assay, *Cry1* knockdown causes a dampened oscillation with a shorter period length, whereas *Cry1^−/−^* murine fibroblast are unable to sustain rhythms. These differences may be due to incomplete *Cry1* mRNA knockdown, the specific workings of U2OS cells versus other types of cells or a combination of both. Nonetheless, it is interesting to note that both the free running activity period of *Cry1^−/−^* mice, as well as that of SCN explants derived from these animals is shortened compared to the wild-type [27], [30]. Overall, this cell-based system is suitable to study the workings of the mammalian circadian oscillator. Furthermore, given that U2OS cells are of human origin, they are an appropriate stand-in in which to study the human clock. Finally, while this work focuses on an siRNA-based method, this format could be adapted for other strategies, such as small-molecule library screening. Altogether, this work contributes to furthering our understanding of the mechanisms of circadian regulation.

## Materials and Methods

### U2OS-B6 cells generation

U2OS cells were transfected with a plasmid harboring a destabilized firefly luciferase (pGL4.22, Promega, Madison, Wisconsin) construct under the control of the mBmal1 promoter (−422 to +108). Stable cells were selected in DMEM plus 10% FBS/1% Penicillin/Streptomycin and 2 and 2 µg/mL puromycin. Clonal cell lines were then established by serial dilution and then tested for the presence of circadian luciferase activity in a 32-channel Lumicycle (Actimetrics).

### Cell Culture and Transfection*s*

U2OS-B6 cells were maintained under standard tissue culture conditions in DMEM (Invitrogen) plus 10%FBS, 1% Penicillin/Streptomycin and 2 µg/mL puromycin. For siRNA transfections, a master mix of Serum- and Phenol Red-free DMEM (7 µL/transfection), HiPerFect reagent (1 µL/transfection, Qiagen) and 2 µL of a 10 µM siRNA solution (2 pmols/transfection) was distributed into each well of a 384-well plate and incubated at room temperature for 20–40 minutes. During this time, U2OS-B6 cells were trypsinized and resuspended in Phenol Red-free DMEM (Gibco, supplemented with 10% FBS, 15 mM HEPES pH 7.4, non-essential amino acids, Sodium Pyruvate and 1% Penicillin/Streptomycin/Antimycotic) to a concentration of 125,000 cells/mL. Following incubation, 40 µL of cells were distributed onto each well. Subsequently, the plates were covered, pulse centrifuged and returned to a water-jacketed 37°C incubator. Forty-eight hours after transfection, cells were shocked for 2 h at 37°C in a final concentration of 100 nM dexamethasone. Following shock, the medium was replaced with Phenol Red-free, supplemented DMEM containing 100 µM D-Luciferin, sealed with a custom-made glass coverslip (#1 borosilicate glass, 0.12–0.16 mm thickness, 115×77 mm area, Erie Scientific, Portsmouth, NH) and subjected to real-time bioluminescence measurements for 5 days.

### siRNAs, Plates, Reader and Data Acquisition

All siRNA duplexes were designed by and purchased from Qiagen (Valencia, CA, sequences provided in Table S1). White, flat-bottom, tissue culture-treated 384-well polystyrol Greiner plates (11500 µm well depth) were read in a temperature-controlled TECAN M200 Luminometer and iTecan Software (Tecan Group, Ltd). The plate height was specified to 14400 µm with a tolerance of 450 µm and luminescence for each well was integrated over 2 seconds and read at 15-minute intervals for five days at a temperature setting of 37°C. Data detrending and smoothing were done in Excel 2007 (Microsoft, Seattle, WA) and subsequently COSOPT analyzed as described in the text.

### QPCR Analysis

Quantitative PCR was performed with ABI SYBR Green 2× Master Mix according to the manufacturer's instructions with the following primers: hBmal1 (F- 5′-CAACCGCAAACGGAAAGGC-3′; R- 5′-ACGCCGCTTTTCAATCTGACT-3′), hCry1 (F- 5′-ACAGGTGGCGATTTTTGCTTC-3′; R- 5′-TCCAAAGGGCTCAGAATCATACT-3′), hActβ (F- 5′-CAT GTA CGT TGC TAT CCA GGC-3′; R- 5′-CTC CTT AAT GTC ACG CAC GAT-3′).

## Supporting Information

Table S1 siRNA duplex sequences in Excel format.
(0.01 MB DOC)
