# Correction: A High-Throughput Assay for siRNA-Based Circadian Screens in Human U2OS Cells

**DOI:** 10.1371/annotation/42c80798-20f2-4a1f-8c1d-290631231cf5

**Published:** 2009-02-12

**Authors:** Christopher Vollmers, Satchidananda Panda, Luciano DiTacchio

In the second sentence of the section "Cell Culture and Transfections" of the Materials and Methods, the concentration of the siRNA solution is incorrect. It should read: "...2 µL of a 1 µM siRNA solution..."

